# Child Welfare

**Published:** 1920-02-21

**Authors:** 


### Child Welfare.

### Women's Share.

The Lord Mayor presided over a meeting at the Mansion House on Monday held by the Children's Era Movement in connection with the National Baby Week Council. Reference was made to the child-welfare movement in Belgium, where juvenile welfare institutions are being reorganised and where the Government have given a loan to organise infant welfare.

Lady Astor, speaking on child welfare and citizenship, said women had a very strong creative instinct and now was their chance to create a new world for the generations that were to come, and especially for the babies which were to be born within the next few years. They wanted an equal chance for every child born into the world to make good physically, mentally, and morally, and if only they could make a clean sweep of drink and disease how much better it would be. It was terrible to think that we were fast losing our gains made during the war, when, thanks to the Liquor Control Board, the evil effects of alcohol were enormously lessened. She mentioned that the number of cases of mothers over-laying their babies was reduced by half during the war, and that the Registrar-General attributed this largely to the control of drink.

People said, give the working man his freedom and his beer. No one wanted to take away that freedom, but they wanted to remove the misery, degradation, and sorrow out of their lives. They had had to face drink in its human aspect, and soon it would have to be faced in its national aspect, and it would be found that so-called temperance cranks were not so cranky as they were imagined to be. Drink was really one of the first things to tackle.

Another urgent question was the provision of better houses, with as many labour-saving—not labour-making—devices as possible. They wanted better facilities for maternity and nursing, more clinics, and more mothers' welfare centres. They wanted the provision of clean milk and more milk for mothers and babies, and for grown-ups too.

Lord Morris spoke of the advantages of giving a good moral education to children from an Imperial point of view, and Mrs. H. A. L. Fisher commended the provisions of the Education Act. The possibilities in regard to the starting of nursery schools under the Act were, in her opinion, among the most important and vital features of the Act.

Lady Astor, at an earlier meeting, made some interesting references to the need for greater representation of women in Parliament to deal with work which demanded women's special attention. Her post-bag, she said, when she first entered the House of Commons, contained 500 letters every day, and it still contained 200 daily. The letters covered a tremendous field, dealing with such subjects as: The adoption of children, the care of the blind, infant welfare and maternity, labour conditions for women, Divorce Law reform, drink, education, food prices, the equal guardianship of children, health and sanitation, housing, hospitals, illegitimate children and their mothers, income-tax hardships, the marriage laws, the endowment of motherhood, milk, penal reform, pension hardships, Poor-Law reform, social purity, tuberculosis, unemployment, international politics, Ireland, Poland, the League of Nations, India, etc.

The mere tabulation of these subjects shows the great field that exists for the wise guidance of women, and there is much to be hoped from the good influence of women in our Legislature by the example and precedent now being so well set by Lady Astor.

